# Decoding disease and therapy through multiomics integration and systems analysis

**DOI:** 10.1093/bib/bbag311

**Published:** 2026-06-14

**Authors:** Mano Joseph Mathew, Joyal Mathew, Ripsy Merrin Chacko, Jagadeesh Bayry, Jean-François Zagury

**Affiliations:** Laboratoire Génomique, Bioinformatique et Chimie Moléculaire, EA7528, Conservatoire National des Arts et Métiers, HESAM Université, 2 Rue Conté, 75003 Paris, Ile de France, France; EFREI Research Lab, Université Paris-Panthéon-Assas, École d’Ingénieurs Généraliste du Numérique - EFREI Paris, 30–32 Avenue de la République, 94800 Villejuif, Ile de France, France; Anhalt University of Applied Sciences, Bernburger Str. 55, 06366 Köthen (Anhalt), Germany; Laboratoire Génomique, Bioinformatique et Chimie Moléculaire, EA7528, Conservatoire National des Arts et Métiers, HESAM Université, 2 Rue Conté, 75003 Paris, Ile de France, France; Integrative Medicine Center, Rafael Institute, 3 Bd Bineau, 92300 Levallois-Perret, Ile de France, France; Hartmann Oncology Radiotherapy Group, Hartmann Radiotherapy Institute, 4 Rue Kléber, 92300 Levallois-Perret, Ile de France, France; Department of Integrative Medicine, Conservatoire National des Arts et Metiers, 292 Rue Saint-Martin, 75003 Paris, Ile de France, France; Institut National de la Santé et de la Recherche Médicale, Centre de Recherche des Cordeliers, Sorbonne Université, Université Paris Cité, 15 Rue de l'École de Médecine, 75006 Paris, Ile de France, France; Department of Biological Sciences and Engineering, Indian Institute of Technology Palakkad, Kanjikode, Palakkad 678623 Kerala, India; Laboratoire Génomique, Bioinformatique et Chimie Moléculaire, EA7528, Conservatoire National des Arts et Métiers, HESAM Université, 2 Rue Conté, 75003 Paris, Ile de France, France

**Keywords:** multiomics, data integration, biomarker discovery, fusion strategies

## Abstract

Computational multiomics methods are based on machine learning methods, and are primarily used for classifying patients into subtypes, discovering novel biomarkers, drug repurposing, and advancing precision medicine. Advances in high-throughput technologies have enabled comprehensive profiling of multiple molecular layers, resulting in the emergence of multiomics approaches for a more accurate understanding of disease mechanisms, therapeutic targets, and biological heterogeneity. This review examines current applications of multiomics in oncology, ageing, and immune-mediated diseases, highlighting the strengths and challenges of integrative models in understanding disease mechanisms, identifying biomarkers, and guiding precision therapies. Integration strategies, from early to late fusion and horizontal to vertical frameworks, are also examined alongside recent advances in computational platforms and preprocessing techniques.

## Introduction

Recent advances in different high-throughput technologies have transformed medical research. The vast amount of data generated by high-throughput technologies such as next-generation sequencing, mass spectrometry, and nuclear magnetic resonance spectroscopy has enabled researchers to better understand the phenomena behind various biological processes and functions [1]. Understanding complex diseases requires more than examining a single layer of biological information. This is because complex diseases such as cancer arise from complex layers of intracellular and intercellular interactions, including transcriptional regulation, gene co-expression, signalling and metabolic pathways, and protein–protein interactions [2, 3]. Apart from these, a tumour is also involved in interactions with noncancerous components such as the tumour microenvironment (TME), the immune system, and gene–environment interactions [4, 5]. The TME plays an important role in regulating and promoting tumour survival by releasing molecules and activating pathways that affect the migratory potential and invasiveness of tumour cells [3]. All these different components have major effect on the tumour growth and possess strong clinical importance for therapy and prognosis. Analysing only one of these levels typically leads to suboptimal or misleading conclusions about disease mechanisms. It is therefore important to investigate these multiple layers of interacting components involved to understand the biological phenomenon.

**Figure 1:**
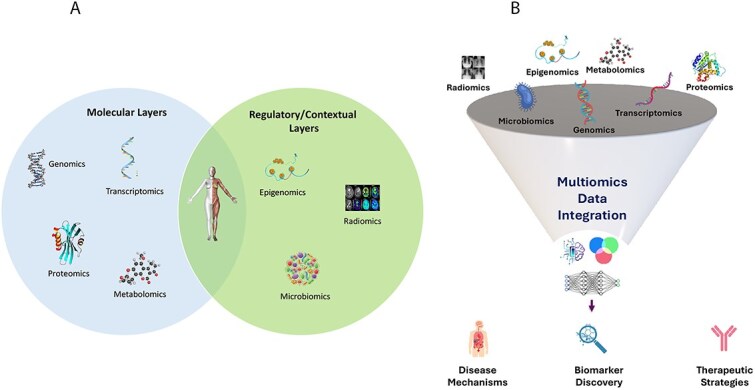
(a) Schematic overview of multiomics data integration. Multiple omics layers including genomics, epigenomics, transcriptomics, proteomics, metabolomics, and microbiomics are combined through integrative approaches. This unified analysis enables the extraction of clinically relevant insights, including the elucidation of disease mechanisms, the discovery of robust biomarkers, and the identification of therapeutic strategies. (b) An overview of multiomics layers. Conceptual organization of multiomics layers. Core molecular layers (genome, transcriptome, proteome, metabolome) and regulatory/contextual layers (epigenome, microbiome, radiome) together represent different dimensions of biological information contributing to phenotype.

Multiomics models uses one or more high-throughput techniques and combines data from multiple omics layers such as genomics, transcriptomics, epigenomics, proteomics, and metabolomics [6]. As shown in Fig. 1A and B, multiple omics layers are combined to obtain relevant insights from multiple biological layers. This provides a deeper understanding of disease biology by combining information from RNA, DNA, proteins, and metabolites. Multiomics enables researchers to follow the molecular processes from gene alteration to phenotypic effects that are often overlooked in single omics research [7]. This systems-level strategy is particularly valuable in complex diseases like cancer, where biological variability and treatment responses can be highly different across patients [8].

While single omics studies have often provided important insights into disease mechanisms, they often fail to present a complete picture of the complex biological processes. For instance, genomic analyses provide information on DNA-level changes and copy number variations but does not capture downstream effects gene expression, protein function, or cellular phenotype [9, 10]. Similarly, transcriptomic data may show variations in gene activity but does not consider the influence of post-transcriptional regulation, protein degradation, or changes in enzymatic activity [11, 12]. Proteomic and metabolic profiles give information on the functional output of the cell, but in the absence of contextual information from upstream regulatory processes, the interpretations become incomplete [13]. Therefore, it is not possible to understand the influence of genetic mutations on cellular behaviour without considering data from multiple molecular levels. Hence, the conclusions from single-omics analyses are often limited and may miss out on crucial mechanisms that contribute to the disease progression, heterogeneity, or therapeutic resistance.

This review focuses on how multiomics helps in understanding disease mechanisms, particularly in the field of oncology. It explores the importance of combining genomic, transcriptomic, proteomic, and other omics layers in identifying therapeutic targets, improving biomarker discovery and in developing effective combination therapies.

### Definitions of omics layers

Multiomics captures biological information across the central dogma from DNA to RNA, proteins, and metabolites. Genomic profiling provides critical insights into mutational burden, driver alterations, and clinically actionable targets [14, 15]. Genomics focuses on the complete DNA sequence and its variations including insertions and deletions (INDELs), single-nucleotide variants, and structural variations such as copy number variations (CNVs) [2]. Transcriptomic data provide insights on gene regulation, phenotype, and cellular type. It records the entire set of RNA molecules expressed in a cell or tissue at a given time [16]. RNA-seq also captures noncoding RNAs such as long-noncoding RNAs (lncRNAs), microRNAs (miRNAs), and small nucleolar RNAs (snoRNAs) [17]. Transcriptomic data also enable researchers to identify dysregulated pathways, aberrant splicing, and immune signatures associated with the disease [18, 19]. Proteomics profiles the complete set of proteins expressed within a biological system giving information about the functional state of cells. Proteomics data also help in identifying dysregulated signalling pathways, neoantigens, and resistance-associated proteins aiding in biomarker discovery [20, 21]. Moreover, it can be used to map dynamic protein changes in response to therapies, which makes proteomics an important tool in drug development [22]. Metabolomics focuses on the comprehensive characterization of metabolites that contributes to or results from cellular metabolism providing information of the biochemical activity and the physiology of the cell [23]. Metabolomics plays a crucial role in understanding metabolic rewiring, a hallmark of cancer, which reflects alterations in glycolysis, amino acid metabolism, and redox state [24, 25].

Apart from core molecular layers, multiomics also capture regulatory and environmental factors that influences cellular states. Epigenomics studies the complete set of epigenetic modifications on the genetic material of a cell. It focuses on the reversible, heritable chemical changes of DNA and histone proteins that control gene expression without changing the nucleotide sequence [26]. The key modifications it focuses include DNA methylation and histone modifications that affect the gene expression without changing the underlying DNA sequence [26]. Microbiomics determines the function and composition of microbial communities using metagenomic techniques such as 16S rRNA sequencing, Metatranscriptomics (MTx), metaproteomics, or shotgun metagenomics (MGx) [27]. Radiomics involves the extraction of high-dimensional quantitative imaging characteristics from clinical imaging modalities such as Computed Tomography (CT), Magnetic resonance imaging (MRI), and Positron emission tomography (PET) to characterize tumour heterogeneity, shape, texture, and intensity patterns Radiomics can also be used to monitor tumour changes over time and guide adaptive treatment strategies. Radiomics is also being increasingly used in cardiovascular disease, pulmonary fibrosis, and neurological disorders, where imaging biomarkers can aid in early diagnosis and risk stratification [28–30].

### Concepts and strategies of multiomics integration

#### Horizontal integration

Multiomics integration strategies are generally classified based on how different types of data are structured and related across biological samples. Horizontal and vertical integration strategies are the two main types of integration strategies used and require different computing approaches.

Horizontal integration involves the integration of the same type of omics data (e.g. transcriptomics or proteomics) from independent datasets, studies, or sets of experiments. Such strategies are necessary in population-level research and large meta-analyses where the aim is to increase statistical power, cross-validating results between cohorts, or improve model generalizability. However, horizontal integration faces significant challenges due to technical heterogeneity, batch effects, variable sequencing depth, and sample quality. Several statistical and machine learning models have been developed to mitigate these issues. ComBat algorithm, which uses an empirical Bayes framework, remains a foundational tool for batch correction [31]. Advanced versions of ComBat algorithms that offers better scalability and performance on complex datasets such as Nested ComBat, OPNested ComBat, ComBat-seq have been developed [32–34].

Harmony and scanorama are two popular tools used in single-cell data integration that projects datasets into a shared low-dimensional space, offering scalable solutions. Harmony has become a popular approach in single-cell transcriptomics for integrating batches of datasets by projecting them into a shared low-dimensional embedding [35]. It has demonstrated good performance in aligning scRNA-seq datasets from various patients and conditions without the overcorrection of biologically interesting signals. Similarly, scVI (single-cell variational inference) employs deep generative modelling with variational autoencoders to represent gene expression distributions, allowing for probabilistic batch correction and imputation [36]. This approach has been effective in creating harmonized atlases across a variety of tissue types and TMEs. Scanorama is another recently developed unsupervised method that performs manifold alignment of single-cell data by matching mutual nearest neighbours across batches [37]. It is particularly suited for the integration of large-scale single-cell RNA-seq datasets with varying levels of sparsity. BBKNN (Batch Balanced *K*-Nearest Neighbours) extends the kNN graph construction by balancing batch contributions, preserving biological variation during clustering and visualization steps in tools like Manifold Approximation and Projection (UMAP) or *t*-distributed stochastic neighbour embedding (*t*-SNE) [38].

**Figure 2:**
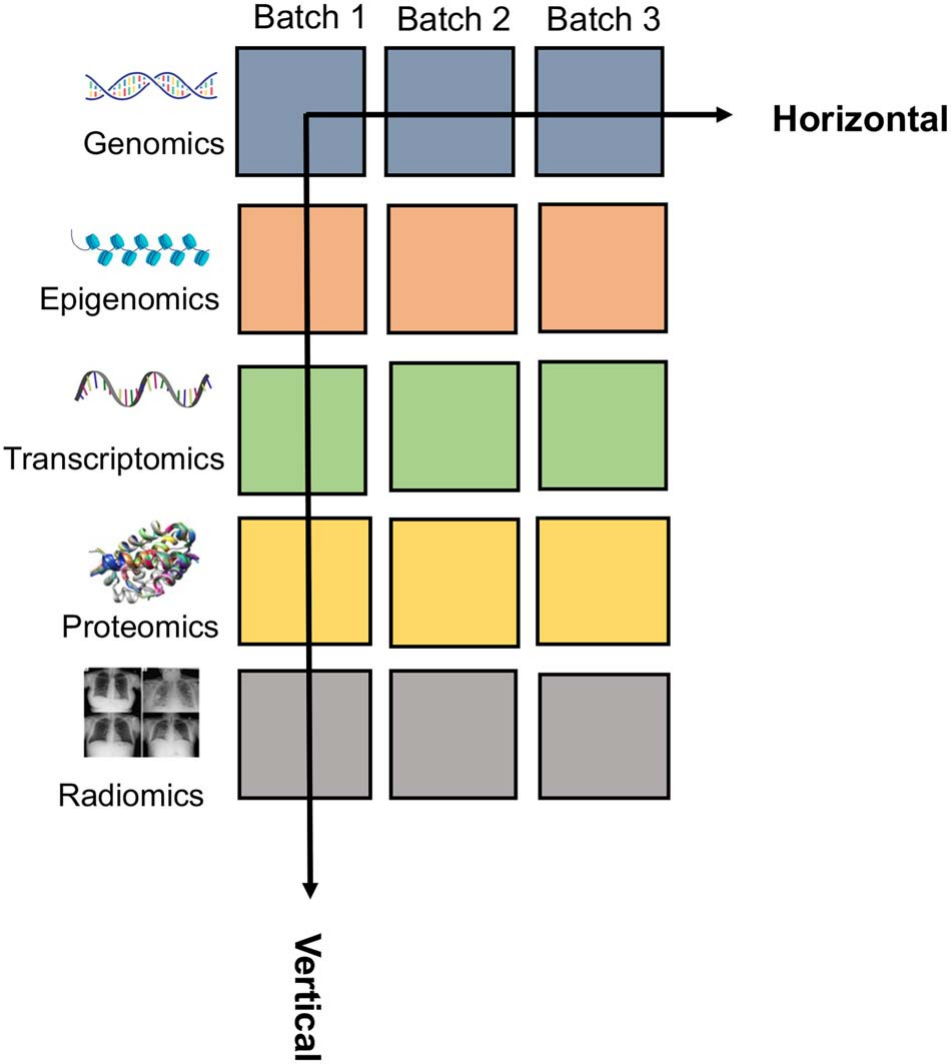
Horizontal versus vertical integration of multiomics data. In horizontal integration, datasets from the same omics type but different batches or cohorts are combined to increase statistical power and reduce batch-specific bias. In vertical integration, multiple omics layers from the same samples (e.g. genomics, epigenomics, transcriptomics, proteomics, radiomics) are integrated to provide cross-modal insights into biological systems and disease mechanisms.

MSstats and Triqler has been used to integrate label-free quantification datasets, correcting for missingness and technical variance in proteomics data [39, 40]. In metabolomics, tools such as Mzmine 3 and XCMS-MELTIN allow alignment and retention time correction across LC-MS batches [41, 42]. QIIME 2 is a popular tool used in microbiomics that supports meta-analyses by harmonizing 16S rRNA gene sequencing data across cohorts with different primers and sequencing depths [43]. These tools broaden the scope of horizontal integration by enabling reproducible analysis across diverse omics modalities. Despite its strengths in increasing statistical power and reproducibility, horizontal integration may be limited when biological variability across cohorts is affected by technical differences, making it difficult to distinguish true biological signals from batch effects. Its effectiveness also depends on the availability of similar datasets with consistent experimental design and metadata annotation. Figure 2 illustrates vertical and horizontal integration methods of multiomics data.

#### Vertical integration

Vertical integration enables simultaneous analysis different types of omics data measured from same samples or subjects. The aim of vertical integration is to understand multi-layered biological regulation and explore cross modal interactions such as how transcription is influenced by genomic alterations, or how metabolic products are influenced by post-translational modification. For instance, vertical integration of phosphoproteomic data, gene expression, and somatic mutation data has identified distinct oncogenic pathways to melanoma subtypes [44].

Vertical integration requires specialized tools that can address the heterogeneous nature of multiomics data. MOFA+ is one such tool that uses a factor analysis formulation to identify common and modality-specific factors across omics types, and imputes missing values [45]. iClusterBayes integrates multiomics by latent variable selection modelling, and its more recent versions available on the iClusterPlus R package are still used for unsupervised subtype discovery of cancer [46, 47]. Vertical integration methods also support a wide range of omics data. DIABLO has gained popularity for integrating transcriptomic, proteomic, and metabolomic layers with built-in visualization and feature selection [48]. Such integrative frameworks are important when analysing complex diseases such as cancer, where regulatory interactions span across the genome, transcriptome, and proteome. While vertical integration provides deeper insight into biological regulation across multiple molecular layers and cross-modal interactions, it is often constrained by the requirement for matched multiomics datasets from the same samples, which can limit sample size and increase data sparsity. Moreover, differences in data scale, noise levels, and missing values across omics layers can complicate integration and interpretation.

### Fusion strategies

Fusion methods determine at what stage processing does the integration of multiomics data takes place. Fusion strategies are typically classified into early, intermediate, and late fusion techniques. These approached differ based on when the data are combined, whether before modelling, during feature transformation, or during decision-making.

#### Early-stage integration

Early-stage integration, or data-level fusion, involves the concatenation of raw or preprocessed feature values across the various omics layers into a composite matrix before downstream analysis. This approach assumes feature comparability across different omics layers either in scale, distribution, or biological meaning, so that they can concatenated into a single matrix and analysed together. But given their intrinsic differences, it is important to perform careful normalization and scaling to make this assumption reasonable for analysis. This method is relatively easier to implement, often employing PCA, *k*-means cluster, or elastic net regression on the combined dataset. Recent developments such as OmiEmbed have introduced deep learning–based frameworks and utilizes unified embedding networks to capture complex nonlinear relationships among concatenated omics features [49]. These models effectively integrate heterogeneous data types and enable better accuracy during phenotype classification, biomarker discovery, and survival prediction by uncovering complex patterns across heterogeneous data types. However, early-stage fusion methods may not be effective when omics features are inherently incompatible in scale, distribution, or biological meaning, leading to distorted representation and reduced model performance. These models also require careful optimization to overcome challenges of high dimensionality, overfitting, and noise inherent to multiomics data.

#### Intermediate-stage integration

**Figure 3:**
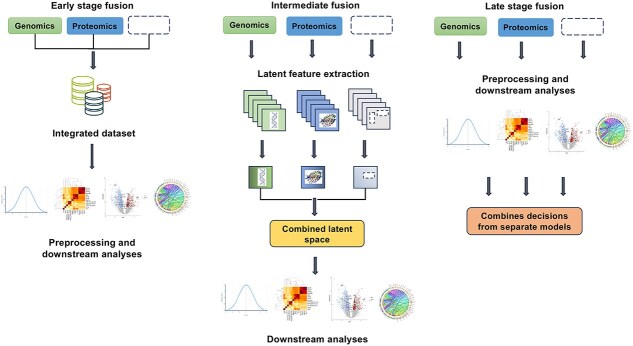
Fusion strategies for multiomics data integration. In early fusion, raw features from multiple omics datasets are combined into a single dataset prior to modelling. In intermediate fusion, each dataset is transformed into latent feature representations, which are subsequently merged for analysis. In late fusion, each dataset is modelled independently, and the resulting predictions are integrated at the decision level. These strategies illustrate how integration can occur at different stages of the analytical pipeline, each with distinct advantages and limitations.

Intermediate-stage integration, also known as transformation-level fusion, processes each omics dataset separately to extract meaningful latent representations and concatenates different layers together before final model building or clustering. Intermediate-level integration balances between the simplicity of early fusion and flexibility to accommodate the heterogeneous nature of multiomics data by not performing direct concatenation of raw features, thereby having the potential to solve problems regarding modality-specific noise and scale differences. Dimensionality reduction techniques such as matrix factorization, canonical correlation analysis (CCA), and manifold learning are commonly used at this stage to project omics data into a common latent space that captures common biological signals without losing modality specific information. Neural network-based methods such as variational autoencoders (VAEs) and graph-based models are now being used to derive reliable low-dimensional representations from each data type prior to integration. DeepMO and GLUER examples of such methods that uses deep learning methods to derive meaningful embeddings from each data type prior to integration [49, 50]. Intermediate integration methods can handle missing data and batch effects better than early-stage fusion and provides clear insights by identifying common and unique components in different omics data. However, intermediate integration can be limited when the learned latent representations fail to capture true biological signals or when important modality specific information is lost during dimensionality reduction. Careful design and testing methods should be used for such models to ensure that the latent features accurately represent the underlying biology rather than the technical artifacts, highlighting the need for reliable computational frameworks and reproducibility in multiomics studies. A schematic representation of early-, intermediate-, and late-stage fusion is illustrated in Fig. 3.

#### Late-stage integration

Late-stage fusion or decision-level fusion involves each modality being analysed separately to produce predictions or intermediate outputs such as risk scores, or clustering labels, which are then fused together using ensemble learning or meta-analysis techniques. When compared to early and intermediate methods that fuse data or latent representations, late fusion emphasizes more on modality-specific interpretability, enabling researchers to maintain the integrity of each omics level while fusing interpretations at a higher decision level. This makes it particularly suitable when the datasets are incomplete, highly heterogeneous, or comes from different cohorts. Commonly used methods in late-stage fusion include majority voting, weighted averaging, and Bayesian model averaging [51, 52]. More recently developed models uses ensemble learning techniques such as stacked generalization, where a meta model is trained to combine predictions from individual models built on separate omics layers [53]. MOLI is an example of one such model that applies late fusion by combining deep neural networks for each omics layer, followed by a logistic regression meta-learner for achieving high performance in predicting drug response using genomic, transcriptomic, and epigenomic data as input [54].

Although late fusion is highly resistant to modality-specific bias and data sparsity, it might lose some of the combined information captured by early and intermediate integration methods. Its accuracy will largely depend on the quality of individual models and the effectiveness of the ensemble model. Yet in cases where datasets are sparse, noisy, or asynchronously obtained, late fusion remains an adaptable and interpretable method, complementary to other integration techniques in the multiomics. However, late fusion may fail to capture cross-modal interactions between different omics layers, as integration occurs only at the decision level rather than at the feature level.

Overall, the choice of integration strategy depends on the characteristics of the data and the analytical objective. Early-stage integration is effective when omics features are comparable and normalized but may fail when feature distributions and biological meanings differ significantly across modalities and is also sensitive to missing data because it requires complete feature matrices. Intermediate integration provides a balance by learning latent representations, making it a more suitable choice for handling heterogeneity and missing data, although its performance depends on the quality and interpretability of the learned embeddings. Late-stage integration is particularly suitable for heterogeneous or incomplete datasets, as it preserves modality-specific information, but may fail to capture cross-modal interactions due to the lack of joint feature level modelling. Therefore, the selection of an appropriate integration strategy should consider factors such as data compatibility, sample size, missing data, and the need for interpretability or cross-modal interaction modelling. A summary of different integration strategies is presented in Table 1.

**Table 1:** Comparison of multiomics integration strategies.

Integration strategy	Description	Pros	Cons	Handling missing data	Best use case
Horizontal	Combine same-omics datasets across cohorts	Increases statistical power	Does not leverage multi-layer interactions	Moderate (depends on preprocessing and batch correction)	Large cohort studies
Vertical	Combine multiple omics from same samples	Captures interlayer biology	Requires matched multiomics data from same subjects	Limited (requires complete matched samples)	Mechanistic studies, pathway and regulatory analysis
Early Fusion	Concatenation before analysis	Simple, captures feature-level synergy	High dimensionality, normalization challenges	Poor (requires complete data matrix)	Small, well-aligned datasets with comparable features
Intermediate Fusion	Dimension reduction then joint modelling	Balance between structure and integration	Model complexity, sensitive to transformations	Moderate (can handle partial missing data)	Heterogeneous datasets with complex relationships
Late Fusion	Independent modelling then result-level integration	Modular; robust per modality	No interaction modelling, less synergy	Good (handles incomplete modalities effectively)	Heterogeneous or partially missing datasets

### Computational pipelines and frameworks

Increased complexity and heterogeneity of multiomics datasets have prompted the development of end-to-end computational pipelines and frameworks that bring together the integration, analysis, and interpretation of various data modalities under a single framework. These platforms not only make it easy to harmonize and preprocess multi-layered omics data but also provide user-friendly environments to implement sophisticated statistical models and machine learning algorithms, enabling researchers to derive biological insights with greater efficiency and reproducibility. Such frameworks typically integrate a combination of statistical methods, machine learning approaches, and deep learning models for processing, integrating, and analysing multiomics data.

Integrative analysis platforms offer support for a broad range of functionalities, including data normalization, batch effect removal, dimension reduction, clustering, network inference, and biomarker discovery. Galaxy, for instance, is an open-source, web-based platform that integrates many bioinformatics software tools into modular workflows, enabling users without extensive programming expertise to perform multi-step analysis on multiple omics levels [55].

Similarly, popular workflow management tools such as Nextflow, Snakemake, and nf-core pipeline collections provide scalable and reproducible solutions for large-scale multiomics data processing [56–58]. These tools enable automation of complex analyses involving multiple steps and support integration of different computational methods with standardized pipelines.

In addition, resources such as LinkedOmics, a curated database plus analysis tool that focuses on integrative analysis of cancer multiomics datasets by providing access to data from The Cancer Genome Atlas (TCGA) alongside visualization and statistical modules [59].

Together, these computational pipelines and frameworks form the backbone of modern multiomics analysis, facilitating high-quality data integration, enhancing interpretability, and accelerating decision-making in a variety of biomedical applications.

Together, these computational pipelines and frameworks form the backbone of modern multiomics analysis, facilitating high-quality data integration, enhancing interpretability, and accelerating decision-making in a variety of biomedical applications. Some of the popular tools and platforms commonly used are summarized in Table 2.

**Table 2:** Representative computational tools and pipelines for multiomics integration.

Tool/platform	Type	Supported omics	Functionality	Key features	Reference
MOFA+	Integration	Transcriptomics, epigenomics, proteomics, metabolomics	Factor analysis for heterogeneous multiomics	Learns shared & modality-specific latent factors; handles missing data	[45]
**DIABLO (mixOmics)**	Integration	Transcriptomics, proteomics, metabolomics	Sparse multivariate integration & feature selection	sparse Partial Least Squares (sPLS)-based integrative modelling; biomarker discovery	[48]
iClusterPlus	Integration	Genomics, transcriptomics, epigenomics, proteomics	Joint latent variable modelling	Integrated clustering; supports mixed data types	[47]
SNF (Similarity Network Fusion)	Integration	Genomics, transcriptomics, epigenomics, proteomics	Network-based data fusion & clustering	Fuses patient similarity graphs from multiple omics	[60]
Harmony	Integration	Single-cell RNA, ATAC, multiomics	Batch correction & integration (mainly single-cell)	Fast shared-embedding alignment across datasets	[35]
Scanorama	Integration	Single-cell RNA-seq	Large-scale single-cell alignment	Manifold alignment; robust to limited cell-type overlap	[61]
TotalVI	Integration	Single-cell RNA + protein (CITE-seq)	Joint modelling of scRNA + protein data	Variational inference; uncertainty-aware latent space	[62]
MultiVI	Integration	Single-cell RNA + ATAC	Multi-modal variational inference	Joint latent representation for discrete & continuous modalities	[63]
OmiEmbed	Integration	Genomics, transcriptomics, epigenomics, proteomics	Deep learning unified embedding	Learns nonlinear joint representations; multi-task (classification/survival)	[49]
CustOmics	Integration	Genomics, transcriptomics, proteomics	Autoencoder-based customizable integration	Flexible architectures; improved prediction & survival modelling	[64]
Galaxy	Pipeline	All major omics (genomics, transcriptomics, proteomics, metabolomics)	Modular workflow management	User-friendly web platform for reproducible multiomics pipelines	[55]
LinkedOmics	Pipeline	Genomics, transcriptomics, proteomics (TCGA/CPTAC)	Cancer-focused database and analysis	Integrates TCGA & CPTAC datasets; enrichment, correlation, and survival modules	[59]
OpenOmics	Pipeline	Genomics, transcriptomics, epigenomics	Cloud-based multiomics analysis	Scalable, high-throughput workflows; collaborative cloud environment	[65]
Seurat v5	Pipeline	Single-cell RNA, ATAC, protein, spatial	Multi-modal single-cell & spatial integration	Weighted nearest neighbour–based framework for RNA, ATAC, protein, and spatial data	[66]

### Data resources for multiomics integration

Publicly available datasets play an important role in enabling integrative analysis by providing access to large-scale datasets across different biological conditions, populations, and disease types. The increasing availability of such datasets has helped improve multiomics research by supporting reproducibility, cross-study comparison, and development of advanced computational models. These datasets allow researchers to combine datasets across different cohorts, validate findings, and train predictive models with improved generalizability. Moreover, standardized repositories help address challenges related to data sharing, annotation, and long-term accessibility, which are essential for advancing collaborative research in systems biology and precision medicine.

Several well-established databases support multiomics research across different domains. Large-scale cancer focused initiatives such as TCGA and the International Cancer Genome Consortium (ICGC) provide comprehensive multiomics datasets, including, genomic, transcriptomic, epigenomic, and clinical data [67, 68]. Public repositories such as the Gene Expression Omnibus (GEO) and ArrayExpress host a wide range of transcriptomic and other omics datasets from various experimental studies [69, 70]. Domain-specific repositories such as the PRIDE and Metabolights provide proteomics and metabolomics data, respectively, offering standardized formats and metadata for large-scale analysis [71, 72]. In addition, single-cell and spatial omics resources, including the Human Cell Atlas and Single Cell Expression Atlas, enable a more detailed analysis of cellular heterogeneity and tissue organization [73, 74]. Platforms such as LinkedOmics further extend these capabilities by integrating multiomics datasets with integrated analysis and visualization tools [59]. Together, these data resources form the foundation for multiomics integration, supporting both methodological development and biological discovery. Commonly used data resources for multiomics data are summarized in Table 3.

**Table 3:** Commonly used data resources for multiomics integration.

Database	Data type	Key features	Reference
TCGA (The Cancer Genome Atlas)	Genomics, transcriptomics, epigenomics, clinical	Large-scale cancer multiomics datasets	[67]
ICGC (International Cancer Genome Consortium)	Genomics, transcriptomics, clinical	International cancer cohort data; cross-population studies	[68]
GEO (Gene Expression Omnibus)	Transcriptomics, epigenomics	Public repository for gene expression data; diverse datasets	[69]
ArrayExpress	Transcriptomics, multiomics	Curated functional genomics data; standardized metadata	[70]
PRIDE	Proteomics	Mass spectrometry–based proteomics datasets	[72]
MetaboLights	Metabolomics	Metabolite profiling data	[71]
Human Cell Atlas	Single-cell omics	High-resolution cellular data across tissues	[73]
Single Cell Expression Atlas	Single-cell transcriptomics	Processed single-cell datasets with visualization tools	[74]
LinkedOmics	Multiomics (cancer-focused)	Integrated TCGA data with analysis and visualization tools	[59]

### Preprocessing in multiomics

Preprocessing and harmonization are important steps in in multiomics integration as they enable meaningful comparisons of biological signals across diverse datasets by effectively minimizing the technical variability. Standard preprocessing steps generally involve normalization, checking for batch effects, missing values imputation, dimensionality reduction, and scaling.

Normalization is performed to rescale the data into a common scale by reducing technical variability and ensuring comparability across samples and different data types. Quantile normalization, median normalization, log transformation, variance-stabilizing transformation (VST) of RNA-seq, and probabilistic quotient normalization (PQN) in metabolomics are some of the commonly used techniques for normalization [75]. Cross-omics normalization methods like feature-specific quantile normalization and ratio-based scaling have been developed to enable joint analysis across heterogenous data [76, 77]. Moreover, newer methods such as Combat-Seq that uses normalization frameworks that incorporate biological covariates and sample-specific variables have shown better preservation of true biological signals [34].

Empirical Bayes methods such as ComBat and its extensions are popular approaches and can remove batch effects while preserving biological variation [78]. For single-cell multiomics, Harmony and scVI have been widely used, employing embedding alignment and probabilistic modeling, respectively [35, 79]. Tools such as MOFA+ integrate batch correction with latent factor modeling to denoise heterogeneous data sets simultaneously [45]. Emerging methods now integrate batch correction with imputation and dimensionality reduction within unified workflows. DeepBID and MIDAS are examples of such integrated methods that uses a deep learning framework for simultaneous denoising, batch handling, and dimensionality reduction of single-cell RNA-seq data [80, 81]. The choice of batch correction method can significantly influence downstream biological conclusions, so careful benchmarking and validation are important.

Missing values in multiomics data occur due to technical detection limits, sample dropout, or incomplete experimental designs or poor tissue quality [82]. Missing data imputation is a crucial step to reduce bias in downstream analysis and enable accurate interpretation of complex biological systems. Imputation methods vary depending on the omics type. In transcriptomics, methods like MAGIC and ALRA use manifold learning and matrix completion to infer missing counts. Deep learning methods are also now being used for missing value imputation in multiomics [83, 84].

Dimensionality reduction reduces the complexity and high dimensionality of multiomics data Classical techniques such as principal component analysis (PCA) and t-SNE are standard, while more recent techniques such as Uniform UMAP are better at preserving local and global structure. Scaling techniques (e.g. z-score scaling, min–max scaling) are applied prior to dimensionality reduction to avoid features with variations in data ranges dominating analyses. Scaling normalizes these differences and ensures comparison across data types [77].

### Multiomics in disease mechanisms

#### From correlation to causation

Unravelling the complexity of disease mechanism requires causal inference across multiple molecular layers beyond simple associations. According to the central dogma of molecular biology, genetic information passes from DNA to RNA to protein and ultimately to phenotype. Evidence suggests that molecular layers closer to the phenotype, such as the proteome, correlate more with traits than transcripts or genetic variants, highlighting the importance of multiple omics layers in causal frameworks [85]. Multiomics facilitates this by integrating from DNA, RNA, protein, and metabolites, enabling reasoning that approximates the flow of causality within biological systems. This shift towards causality is also evident in network-based computational models like CausalPath, integrating proteomic changes and prior knowledge from pathway databases to infer directed causal paths between perturbations and downstream responses [86]. Similarly, probabilistic graphical models like Bayesian networks and structural equation models are being increasingly used with multiomics data to model probabilistic dependencies and infer directional effects between molecular variables, even under high dimensionality [87, 88].

Recent methodological advances have also introduced graph-based deep learning architectures such as GraphSurv, LAGProg, MoGCN, MOGAT, and MOGONet that model multiomics features on gene or patient similarity graphs to learn causal relationships for predicting clinical outcomes and disease subtypes [89–93]. In addition, recent advancements have explored multi-task graph learning frameworks to jointly model relationships between different molecular entities, such as lncRNAs, miRNAs, and diseases, improving prediction performance through shared representations across multiple related predictions [94]. Graph-based deep learning models use Graph Convolutional Networks (GCNs) to improve classification accuracy for disease subtyping, diagnosis, and identification of new targets or driver genes [95]. These models typically present multiomics data as interconnected graphs, where nodes represent genes or samples and edges capture the biological relationships or similarity. For instance, models such as MoGCN and MOGONet use GCNs to learn modality-specific representations from each omics layer and integrate them using a supervised classification model for patient classification and biomarker prediction [89, 92]. Other models such as MOGAT extend this approach by using attention mechanisms to weight the relative importance of different omics modalities during integration [91]. Frameworks like GraphSurv use graphical representations to identify patient-level relationships for predicting a patient’s survival rate [90]. These supervised models generally outperform purely correlational ones by taking advantage of structured dependencies due to known pathways or network topology inference.

In addition to correlation-based methods, causal inference methods such as Mendelian randomization are also used in multiomics research to infer effects from genetic variation to downstream molecular and clinical phenotypes [96].

#### Multiomics in cancer

Recent multiomics studies have transformed our understanding of cancer biology by enabling integration and analyses of diverse molecular layers to yield actionable insights.

The Pan-Cancer Analysis of Whole Genomes (PCAWG) is considered as a landmark study, integrating whole genome and transcriptomic profiles of metastatic and primary tumours in 38 cancer types [67]. The study revealed that metastatic tumours exhibit a distinct structural variant and clonal mutation compared to primary tumours, offering insights into disease progression. These findings highlight the ability of pan-cancer multiomics approaches to distinguish tissue-specific and shared molecular mechanisms. The Clinical Proteomic Tumour Analysis Consortium (CPTAC) study on colon cancer, using whole-exome sequencing, transcriptomics, global proteomics, and phosphoproteomics data, identified the correlation between Rb phosphorylation and increased proliferation, while microsatellite instability-high (MSI-H) tumours exhibit glycolytically driven immune evasion by suppressing CD8+ T-cell activity [97]. These observations highlight the potential of targeting metabolic pathways and kinase inhibitors to improve outcomes in specific patient subsets.

The emergence of spatial transcriptomics has further enriched the context by introducing a spatial dimension to molecular profiling. In a recent study on hepatocellular carcinoma (HCC), combining scRNA-seq and spatial transcriptomics showed overexpression of PRKDC in immune-excluded tumour regions [98]. Functional assay revealed PRKDC’s role in cell proliferation, making it a potential prognostic biomarker and therapeutic target. Another study using spatial single-cell multiomics on HCC identified a specific stromal fibroblast subpopulation marked by COL1A2, COL4A2, CTGF, and FSTL1 [99]. These cancer-associated fibroblasts were found to promote aggressive growth and were associated with poor prognosis.

Melanoma, characterized by its high immunogenicity and therapeutic heterogeneity has been widely studied using multiomics approaches. An integrated single cell and spatial transcriptomic analysis of acral melanoma found an association of MYC+ tumour cells and fatty acid oxidation–driven metabolism in localized lymph node metastasis, identifying cell state and metabolic dependencies that lead to dissemination [100]. Another multiomics analysis of 68 melanoma cell lines integrating genomics, methylation, RNA, miRNA, lncRNA, and proteomic data revealed how intrinsic cell-state phenotypes are regulated through complex multi-layer regulatory networks, linking phenotypic plasticity to variable drug resistance and treatment outcomes [101]. Furthermore, a multiomics study comparing melanoma brain metastases to extracranial lesions showed an enrichment of oxidative phosphorylation pathways and lower immunogenicity in the brain microenvironment, revealing the site-specific adaptations in metastatic melanoma [102].

Similar studies were also found to be effective in many types of cancers, including breast and lung cancers, as well as rare malignancies such as skull-base chordoma, and is summarized in Table 4. These studies highlight the effectiveness of multiomics studies in understanding disease mechanisms in oncology.

**Table 4:** Applications of multiomics in cancer: insights from recent studies.

Disease	Omics layers	Key findings	Reference
Pan-cancer (PCAWG, 38 tumour types)	WGS, RNA-seq	Metastatic tumours show distinct structural variants and clonal evolution pathways	[67]
Colon cancer (CPTAC proteogenomics)	WES, RNA, proteomics, phosphoproteomics	Rb phosphorylation and metabolic rewiring linked to immune exclusion and aggressiveness	[97]
Hepatocellular carcinoma (HCC)	scRNA-seq + spatial transcriptomics	PRKDC overexpressed in immune-excluded tumour niches	[98]
Liver (HCC)	Single-cell & spatial transcriptomics	F5-CAF fibroblast subset promotes stemness and adversity via niche interactions	[99]
Breast cancer—difficult-to-treat basal-like (DTBC)	WGS, RNA-seq, global proteomics, phosphoproteomics	High-relapse DTBC shows 1q21 amplification and phosphoproteomic signatures linked to proliferation and signaling rewiring.	[103]
Melanoma	scRNA + spatial transcriptomics	MYC+ melanoma cells and FAO metabolism linked to lymph node metastasis niches	[100]
Melanoma	WES, transcriptome, miRNA, lncRNA, methylation, RPPA	Distinct intrinsic phenotypic states controlled by multi-layer regulation, tied to therapy response patterns	[101]
Melanoma brain metastases	Genomics, transcriptomics, proteomics	Brain metastases enriched for oxidative phosphorylation and display reduced immunogenicity	[102]

#### Multiomics in aging research

Multiomics approaches are now also increasingly used in ageing research. Ageing is a time-dependent physiological process that is characterized by multiple biological layers. Multiomics studies provide a comprehensive view of how molecular changes across multiple omics layers affect ageing, uncovering tissue- and cell-type-specific patterns that are often missed by single omics analyses.

A recent study by analyzing proteomic, metabolomic, and methylomic profiles from multiple cohorts over time reported that ageing progresses in a nonlinear manner, with each omics layer exhibiting distinct transition points that single-layer ageing clocks fail to detect [104]. Multiomics ageing clocks that integrate transcriptomic, epigenetic, proteomic, and metabolic data outperform single-omics models by capturing complementary biological hallmarks, resulting in improved accuracy and generalization across diverse populations and tissues [104, 105].

Single cell multiomics has transformed ageing research by providing a granular-level view of cellular changes that occur over time. For instance, a recent study combining single-nucleus RNA seq and ATAC-seq data mapped how ageing affects human skeletal muscles and created the first atlas of cellular senescence in ageing human skeletal muscle [106]. Multiomics has also enabled mapping of molecular ageing across multiple tissues. A cross-tissue multiomics study combining proteomic and metabolomic profiling across 21 organs in mice have identified both tissue-specific and shared molecular remodelling during ageing [107]. The findings reveal disruption of coordinated molecular pathways, with alterations in complement activation, lipid metabolism, and immune regulation, providing a systems-level view of ageing in different organs.

Together, these studies highlight the contribution of multiomics studies in unravelling the complexity of ageing. A selection of recent high-impact studies including those already mentioned is summarized in Table 5.

**Table 5:** Multiomics studies in aging: models, modalities, and molecular insights from recent studies.

Model/cohort	Omics layers	Key findings	Reference
Human longitudinal cohorts (multi-cohort)	Proteome, metabolome, methylome, clinical	Multiomics revealed nonlinear ageing patterns with each omics layer showing distinct transition points.	[104]
Human immune landscape across lifespan	scRNA-seq, TCR/BCR sequencing, mass cytometry proteomics	Elucidates distinct ageing patterns across T- and B-cell subsets, trajectories in clonality, and protein markers, enabling an immune-age prediction model based on integrated omics	[108]
Cross-tissue mouse atlas of ageing (21 organs)	Proteome, metabolome, metagenomic (cross-tissue)	Tissue-specific metabolic and protein remodelling disrupt coordinated pathways across organs	[107]
Population cohorts (UK Biobank-scale multi-omics on ageing & longevity)	Genomics, transcriptomics, metabolomics	Ageing and longevity driven by distinct biological pathways beyond single-omics signals	[109]
Longitudinal human proteome (3 time points, 9 years)	Proteome + clinical traits	Longitudinal changes in serum proteins identify biomarkers linked to age-related chronic disease risk	[110]
Human skeletal muscle ageing (single-cell multiome atlas)	snRNA-seq + snATAC-seq	Defined a cellular senescence atlas in ageing skeletal muscle, revealing heterogeneity in SASP regulation.	[106]

#### Multiomics in immunology

The immune system is highly complex and exhibits high cellular heterogeneity, dynamic signaling networks, and metabolic plasticity, making it difficult for single omics approaches to capture [111, 112]. Multiomics approaches integrate multiple omics layers at both bulk and single-cell resolution, enabling detailed insights into immune regulation mechanisms.

A multi layered analysis using scRNA seq, ATAC-seq, spatial transcriptomics, and imaging data helped reveal the microenvironment of the inflamed synovium at an unprecedented level [113]. While transcriptomic profiling alone can identify types of immune cells, this multiomics approach revealed the distinct epigenetic landscapes of fibroblast-like synoviocytes (FLSs) that shape their inflammatory behaviour. The study identified key FLS subsets that drive pathogenic immune and stromal interactions by connecting chromatin accessibility patterns with spatial transcriptional profiles, providing insights that would otherwise remain elusive with single omics analyses.

A study by Mennillo *et al*. [114] combined single-cell and spatial multiomics to investigate the effects of anti-integrin therapy in inflammatory bowel disease (IBD). The study revealed that response to the therapy correlated with shifts in mononuclear phagocyte and fibroblast populations and their spatial organization, demonstrating how tissue microenvironments also play a role in therapeutic resistance. Such findings highlight the ability of multiomics studies to capture intercompartment interactions that single-cell transcriptomics alone could overlook. Similar studies were also performed on Covid-19. An integrated study on Covid-19 using proteomics, metabolomics, and transcriptomics data revealed disruptions of central metabolism and mitochondrial pathways correlated with disease severity [115].

The strength of multiomics lies in its capability to integrate multiple molecular levels while simultaneously capturing the diversity of cells and their interaction within the microenvironment. This dual perspective offers a more comprehensive framework to understand immunological diseases. Some of the recent studies that effectively used multiomics to understand the disease mechanisms in immune mediated diseases are summarized in Table 6.

**Table 6:** Multiomics in immune-mediated diseases: disease-specific insights from integrated analyses.

Disease	Omics layers	Key findings	Reference
Rheumatoid arthritis (RA) synovium	scRNA-seq + scATAC-seq + spatial transcriptomics + multiplexed imaging	Reveal pathogenic fibroblast-like synoviocyte (FLS) subsets driving inflammatory circuits.	[113]
Inflammatory bowel disease (IBD)	Single-cell & spatial multi-omics	Shows how stromal, epithelial, and immune compartments reorganize under anti-integrin therapy, explaining response versus nonresponse	[114]
COVID-19 (severity stratification)	Proteomics + metabolomics + transcriptomics (multi-omics networks)	Progressive disruption of central metabolism mechanistically tracks with disease severity	[115]
Multiple sclerosis (MS)	Genetics + plasma & brain proteomics (multi-omics causal integration)	Prioritizes 18 proteins with potential causal roles in MS by integrating multi-omics and causal inference	[116]
Systemic lupus erythematosus (SLE)	Transcriptomics + metabolomics + (immune) proteomics/lipidomics (integrative)	Integrative B-cell and systemic profiling reveal metabolic changes linked to SLE activity	[108]

#### Multiomics in systems vaccinology

The advent of multiomics systems vaccinology integrating transcriptomics, proteomics, metabolomics, cytometry, and other high-dimensional modalities has enabled the delineation of early molecular signatures that correlate with vaccine-induced immune outcomes (e.g. magnitude of antibody- or T-cell response) across diverse platforms [117, 118]. In a systems vaccinology study of H5N1 + AS03 vaccination in humans, a platelet- and adhesion-related transcriptional signature at Day 7 postvaccination was predictive of antibody durability (i.e. longer-term maintenance) across individuals [119]. Furthermore, a recent ‘universal signature’ across 13 different vaccines was shown to forecast the magnitude of antibody induction, setting the stage for pan-vaccine predictive biomarkers [120]. Multiomics resources such as the CMI-PB dataset (Tdap booster context) facilitate benchmarking predictive modelling approaches by providing matched baseline and postvaccine omics data across modalities [121]. Finally, methodological advances like RISE (Rank-based Identification of high-dimensional Surrogate Markers) address the challenge of inferring longitudinal immunity (e.g. antibody titres at late timepoints) from early omics measurements and may help flag nonresponders or subjects with waning responses [122]. These findings offered a systems-level perspective, enabling more accurate patient stratification.

#### Multiomics in identification of biomarkers and design of novel therapies

The development of effective therapies depends on the detailed understanding of molecular mechanisms driving the disease. Traditional single-omics approaches only provide a limited knowledge of the complex biological systems involved. Multiomics integration not only helps in understanding the complex disease mechanisms involved but also provides novel insights into drug response mechanisms and aids in revealing new therapeutic targets and pathways. Compared to single-omics approaches, multiomics approaches enables researchers to identify the molecular changes and the drivers of those changes, paving the way for precision diagnostics and targeted therapies in various diseases.

In oncology, disease heterogeneity and therapy resistance often undermine the predictive value of individual biomarkers. Predictive models integrating multiple omics layers perform better than those solely based on a single layer, enabling accurate patient categorization and guiding the selection of drug combinations targeted to specific disease subtypes. A recent multiomics study by Chen *et al*. on metastatic melanoma patients characterize pyroptosis-related signalling modules that classify immunotherapy responder and nonresponder groups [123]. The study predicted response beyond T-cell infiltration markers alone, pointing to pyroptosis and metabolic inflammation as a target for therapeutic intervention. Similarly, another integrated study on melanoma defined adenosine-signalling program predictive of immune checkpoint inhibitor responsiveness [124]. The adenosine pathway was found to represent a checkpoint responsive to therapeutic modulation, as a potential biomarker and providing insights into resistance pathways. In head and neck squamous cell carcinoma, integrated analysis of genomics, copy number variation, DNA methylation, and transcriptomics found three different molecular subtypes with distinct genomic features and TME compositions. Notably, one of the subtypes was characterized by activated epidermal growth factor receptor (EGFR) signalling and an inflamed microenvironment with cancer-associated fibroblast and vascular infiltration, demonstrating sensitivity to EGFR inhibitors. Such integrative studies enable efficient patient subtype classification and targeted therapeutic intervention.

Multiomics approaches are also being increasingly applied in ageing research and immunology for biomarker identification and drug design. In a longitudinal study of ageing individuals by combining proteomic, metabolic, and methylation profiles suggested that molecular changes occur in distinct phases, with major changes happening around midlife and in later stages [104]. These changes were linked with serious health issues such as heart and kidney disorders, and the findings offered insights into biomarkers that correlates with ageing and chronic disease. In a randomized placebo-controlled trial, multiomics analysis found reduced biological age in older adults when treated with therapeutic plasma exchange combined with intravenous immunoglobulin (IVIG) [125, 126]. Similarly, in immune mediated diseases, multiomics has proven effective in identifying targets and predicting therapeutic responses. In Crohn’s disease, baseline gut microbiome and bile acid metabolism profiles predicted responses to anti-α4β7-integrin therapy. Increased abundance of secondary bile acid–producing microbes (Clostridium, Lactobacillus) and elevated levels of secondary bile acids were predictive of good outcomes [127]. Such multi-omics biomarkers not only outperform conventional inflammatory markers but also implicate the microbial–metabolite–receptor axis (FXR/TGR5) as a mechanistic target for probiotic or metabolic therapies.

Multiomics approaches are redefining biomarker discovery and drug design across various disease domains. These approaches move beyond mere correlation by connecting the markers with causal pathways or regulatory mechanisms, providing deeper understandings that can guide targeted interventions. Table 7 summarizes some of the recent studies that used multiomics approaches to identify novel markers and develop new therapeutic combinations. These examples highlight the impact of multiomics frameworks identifying targets and in providing personalized care. As technologies advance and datasets grow, multiomics is poised to become a central pillar of precision medicine.

**Table 7:** Recent applications of multiomics in biomarker discovery and therapy design.

Disease/focus	Omics layers	Key findings	Type	Reference
Head & neck SCC (cancer therapy stratification)	Genomics, CNVs, methylome, transcriptome	Defines molecular subtypes with distinct therapeutic vulnerabilities (e.g. EGFRi sensitivity)	Therapy Design	[128]
Metastatic melanoma (pyroptosis & immune microenvironment)	Transcriptomics, proteomics, metabolomics	Identifies pyroptosis-related signalling modules predictive of immunotherapy response	Biomarker Discovery	[123]
Melanoma tumour ecosystem (adenosine signalling)	Transcriptomics, proteomics, metabolomics	Adenosine pathway signalling identified as predictive of immunotherapy benefit	Biomarker Discovery	[124]
Difficult-to-treat breast cancer (DTBC)	Whole-genome sequencing, transcriptomics, global proteomics, phosphoproteomics	Integrated proteogenomic profiling identifies relapse-risk subtypes and proliferation-associated vulnerabilities in difficult-to-treat breast cancer.	Biomarker Discovery/Therapy	[103]
Ageing cohort (longitudinal proteome + clinical)	Proteome, clinical	Identifies serum proteome ageing score predictive of cardiometabolic disease risk	Biomarker Discovery	[110]
Lung adenocarcinoma and squamous NSCLC	Genomics, transcriptomics, proteomics, phosphoproteomics	Five subtypes identified; proliferative subtype with PI3K–Akt activity and Whole-Genome Doubling linked to poor survival; phosphoproteomics reveals signalling and metastatic drivers.	Biomarker Discovery/Therapy	[129]
Cervical cancer (plasma proteomics)	Targeted proteomics (Olink panel)	Five-protein signature predictive of immunotherapy response and prognosis	Biomarker Discovery	[130]
COVID-19 immunopathology	Plasma proteomics + metabolomics, transcriptomics	Multiomics networks decouple metabolic versus inflammatory drivers of severity, guiding therapy	Biomarker Discovery/Therapy	[131]
IBD integrin therapy response	Whole-genome metagenomics, metabolomics	Microbiome-mediated bile acid metabolism predicts response to anti-α4β7 therapy	Biomarker Discovery	[127]
Ageing rejuvenation interventions	Transcriptome + methylome + proteome	Multiomics clocks detect coordinated biological age reversal postplasma exchange + IVIG	Biomarker Discovery/Therapy	[125]

## Discussion

### Limitations and current challenges in multiomics integration

Despite the growing success of multiomics in understanding disease mechanisms and guiding precision medicine, several challenges still hinder their broader application. These limitations arise from both computational and biological aspects. One of the major challenges is the technical and analytical complexity in integrating heterogenous data types from various platforms. Each omics layer has unique characteristics in terms of dimensionality and scale. Integrating these different layers without losing biological signal is highly important. While early-stage integration can oversimplify relationships, intermediate- and late-stage integration machine learning or graphical models require considerable computational knowledge and resources.

Multiomics data are often generated from various experimental platforms, laboratories, and protocols, resulting in batch effects, scale discrepancies, and missing data that cause challenges in downstream analyses. Addressing these challenges are essential for ensuring the integrity and comparability of multiomics data prior to integration. Various preprocessing methods required increases the computational complexity of integrating multiomics data. Each omics modality presents its own preprocessing challenges. For instance, transcriptomics count data require normalization for sequencing depth and gene length, proteomics data must be adjusted for batch-specific biases, and metabolomics data often require scaling to account for large variations in metabolite concentrations.

Sample heterogeneity and small cohort sizes also pose significant challenges. Generating high-quality multiomics datasets from the same sample is technically demanding and expensive, often requiring compromises in resolution or coverage. A smaller cohort size reduces the statistical power of the study and increases the likelihood of false positives or overfitting. Smaller sample size also makes it difficult to capture the biological heterogeneity of the disease.

Moreover, batch effects and platform biases might be mistaken as biological variation, requiring normalization and corrections. Batch effects are caused by nonbiological variation due to differences in sample processing times, reagents, or equipment and can hide true biological signals leading to inaccurate conclusions. Correcting batch effects is particularly challenging in multiomics studies where each omics type may have distinct batch structures. High dimensionality and presence of missing data also increase the computational complexity involved in integrating multiomics data. Missing value imputation has shown to improve accuracy in downstream analyses. Despite advances, imputing missing values remains a challenge when the proportion of missing data is high and is varied across different omics datasets [82].

Lack of end-to-end computational pipelines and standardized workflows is another major bottleneck in multiomics integration. Most analysis are performed on custom-built pipelines that leads to poor reproducibility and comparability across studies. Absence of automated pipelines makes it difficult to handle high-dimensional datasets, slowing down analysis and limiting accessibility for noncomputational researchers.

Biologically, another concern is sparse functional annotation of multiomics correlations, highlighting the need for approaches that go beyond simple correlations and focus on uncovering true biological mechanisms, such as casual inference models. Current integration methods continue to face persistent technical challenges, including limited scalability, reduced interpretability, and poor generalizability across independent datasets. Furthermore, the absence of standardized benchmarking frameworks hinders objective and reproducible comparison of model performance. Although existing computational platforms can effectively identify patterns across multiple data layers, the mechanistic and causal interpretation of detected associations remains uncertain without complementary experimental validation. This uncertainty substantially restricts the translational relevance of multiomics findings in clinical contexts. To address these limitations, future research should prioritize the incorporation of explainable artificial intelligence, network-based modelling approaches, and rigorous experimental validation strategies. Specifically, adopting causal inference frameworks will enable researchers to transition from purely correlative analyses towards more biologically meaningful and clinically actionable interpretations. Collectively, resolving these methodological and biological challenges is critical for enhancing the reliability, reproducibility, and translational applicability of multiomics research while simultaneously defining key priorities for advancing the field.

### Future directions

The future of multiomics is likely to be determined by several converging developments. The application of deep learning techniques is expected to improve pattern recognition in complex multilayered datasets. Emerging techniques such as digital twins, causal inference models aimed at improving mechanistic interpretation, and temporal multiomics are showing promise in dynamic disease modelling and optimizing therapy. Experimentally, the advent of ultra-sensitive multiomics profiling of low-input samples such as single-cell multiomics or spatial-omics will enable us to improve tissue microenvironment understanding and cellular states.

Importantly, multiomics also holds immediate translational relevance. Integrative and personalized medicine could be enhanced by multiomics via tailoring interventions to individual molecular profiles [132]. This approach enables practitioners to select conventional or complementary therapies based on a patient’s unique genetic, proteomic, and metabolic signatures, thereby optimizing outcomes and minimizing adverse effects. In oncology, for instance, multiomics-driven biomarker panels already guide drug selection and monitor therapeutic efficacy [133, 134].

Besides, initiatives such as the Human Cell Atlas and TCGA Pan-Cancer Atlas are laying the foundation for open-access, harmonized multiomics datasets for cross-cohort meta-analyses and accelerating discovery. Eventually, the full potential of multiomics will be achieved through not only technological development but also deliberate effort towards standardization, multidisciplinary collaboration, and equitable data sharing. More recently, large language models (LLMs) have emerged as a promising tool in multiomics research. These models can integrate heterogeneous biomedical data with textual knowledge from scientific literature, enabling improved interpretation of complex biological relationships [135]. LLMs also facilitate automated hypothesis generation, data annotation, and knowledge extraction, offering a new paradigm for integrating omics data with clinical and biological insights [135]. However, challenges such as interpretability, data bias, and validation remain to be addressed before widespread adoption.

## Conclusion

Multiomics has evolved from a conceptual framework into a practical approach, improving our ability to understand the molecular architecture of complex diseases. By integrating heterogeneous layers of genomics, transcriptomics, proteomics, metabolomics, epigenomics, and medical imaging, it overcomes the limitations of single-omics approaches and provides a better understanding of disease heterogeneity, progression, and treatment response. However, current integration strategies have many limitations. Early integration captures interactions between various data types but is limited by dimensionality and noisy data. Intermediate approaches, including network-based and latent factor models such as MOFA+ and similarity network fusion, preserve the modality-specific structure and are easier to interpret, yet remain sensitive to batch effects. Late-stage integration is more flexible and handles missing data better but may overlook interactions between different omics layers.

Deep learning methods, particularly graph neural networks, are now more widely used and can capture complex patterns in multiomics data. However, their limited interpretability remains a major challenge for clinical adoption, leading to growing interest in explainable models that incorporate biological knowledge.

Several challenges still remain, including differences between datasets from different platforms, lack of standardized preprocessing methods, and the computational demands of large and sparse datasets such as single cell and spatial data. However, new developments in spatial and single-cell multiomics are helping to address these challenges by providing better understanding of tissue organization and cellular features. In addition, single-cell multimodal assays such as CITE seq and 10× Multiome enable the simultaneous profiling of regulatory and phenotypic states within the same cell, while approaches such as federated learning support data analysis across institutions without compromising on data privacy. Moreover, the integration of multiomics with artificial intelligence, digital pathology, and clinical data is enabling the development of patient-specific digital twins and predictive disease modelling.

Looking ahead, progress in multiomics will depend on better data standardization, scalable computational frameworks, and improved model interpretability. With these developments, multiomics is likely to play an important role in biomarker discovery, therapy development, and precision medicine.

**Key Points**

- We review current multiomics technologies and integration strategies across genomics, transcriptomics, proteomics, metabolomics, and other regulatory layers, providing a systems-level perspective of disease biology.
- The article covers major computational frameworks for multiomics integration, including statistical models, network-based approaches, and machine learning methods for extracting mechanistic and clinically relevant insights.
- Representative applications demonstrating how multiomics has been used in decoding disease mechanisms, refining molecular subtypes, identifying biomarkers, and enabling therapeutic discoveries across cancer, ageing, and immunological disorders is discussed.
- Finally, we highlight the current challenges, including data heterogeneity, computational complexity, and causal inference limitations, and discuss future directions involving single-cell, spatial multiomics, and advanced integrative modeling approaches.

## Data Availability

No new data were generated or analysed in support of this research.
